# Dr. J. K. Walther

**Published:** 1858-05

**Authors:** 


DR. J. K. WALTHER, one of the oldest and most distinguished members of the University of Leipzig, died on the 4th of March, at an advanced age. He was the author of a number of works upon surgery, among others, one on Crural Hernia, which made his name universally known to scientific men.

